# Immune-Stromal Score Signature: Novel Prognostic Tool of the Tumor Microenvironment in Lung Adenocarcinoma

**DOI:** 10.3389/fonc.2020.541330

**Published:** 2020-09-23

**Authors:** Xiaoguang Qi, Chunyan Qi, Boyu Qin, Xindan Kang, Yi Hu, Weidong Han

**Affiliations:** ^1^Department of Oncology, Chinese PLA General Hospital, Beijing, China; ^2^Department of Health Management, Chinese PLA General Hospital, Beijing, China; ^3^Department of Bio-therapeutic, Chinese PLA General Hospital, Beijing, China

**Keywords:** tumor microenvironment, lung adenocarcinoma, prognostic model, TCGA database, ESTIMATE algorithm

## Abstract

**Background:** Immune and stromal cells in the tumor microenvironment (TME) significantly contribute to the prognosis of lung adenocarcinoma; however, the TME-related immune prognostic signature is unknown. The aim of this study was to develop a novel immune prognostic model of the TME in lung adenocarcinoma.

**Methods:** First, the immune and stromal scores among lung adenocarcinoma patients were determined using the ESTIMATE algorithm in accordance with The Cancer Genome Atlas (TCGA) database. Differentially expressed immune-related genes (IRGs) between high and low immune/stromal score groups were analyzed, and a univariate Cox regression analysis was performed to identify IRGs significantly correlated with overall survival (OS) among patients with lung adenocarcinoma. Furthermore, a least absolute shrinkage and selection operator (LASSO) regression analysis was performed to generate TME-related immune prognostic signatures. Gene set enrichment analysis was performed to analyze the mechanisms underlying these immune prognostic signatures. Finally, the functions of hub IRGs were further analyzed to delineate the potential prognostic mechanisms in comprehensive TCGA datasets.

**Results:** In total, 702 intersecting differentially expressed IRGs (589 upregulated and 113 downregulated) were screened. Univariate Cox regression analysis revealed that 58 significant differentially expressed IRGs were correlated with patient prognosis in the training cohort, of which three IRGs (*CLEC17A, INHA*, and *XIRP1*) were identified through LASSO regression analysis. A robust prognostic model was generated on the basis of this three-IRG signature. Furthermore, functional enrichment analysis of the high-risk-score group was performed primarily on the basis of metabolic pathways, whereas analysis of the low-risk-score group was performed primarily on the basis of immunoregulation and immune cell activation. Finally, hub IRGs *CLEC17A, INHA*, and *XIRP1* were considered novel prognostic biomarkers for lung adenocarcinoma. These hub genes had different mutation frequencies and forms in lung adenocarcinoma and participated in different signaling pathways. More importantly, these hub genes were significantly correlated with the infiltration of CD4+ T cells, CD8+ T cells, macrophages, B cells, and neutrophils.

**Conclusions:** The robust novel TME-related immune prognostic signature effectively predicted the prognosis of patients with lung adenocarcinoma. Further studies are required to further elucidate the regulatory mechanisms of these hub IRGs in the TME and to develop new treatment strategies.

## Introduction

Lung cancer is still the leading disease worldwide in terms of the threat to human life and health (1, 2), and lung adenocarcinoma is the most common pathological subtype. Studies in the past decade have reported that tyrosine kinase inhibitors (TKIs) targeting epidermal growth factor receptor (EGFR), anaplastic lymphoma kinase (ALK), and ROS proto-oncogene 1 (ROS1) are potential therapeutic targets for lung adenocarcinoma, upon genotyping (3–5). Molecular-targeted therapy based on these sensitive targets has considerably enhanced overall survival (OS) among patients with lung adenocarcinoma; however, this therapy is not suitable for all patients with lung adenocarcinoma. Furthermore, drug resistance is common among patients receiving molecular-targeted therapy, and their prognosis is poor (6, 7). Nonetheless, numerous studies have led to the advancement of immunotherapy for several cancers, including lung adenocarcinoma. Immunotherapy is different from targeted therapy; it has more durable clinical benefits. Furthermore, some antibodies used for immunotherapy have been successfully approved as first- and second-line treatments for advanced lung adenocarcinoma (8). In particular, the immune system reportedly plays an important role in the pathogenesis and prognosis of lung adenocarcinoma (9). Therefore, it is essential to understand the immune prognostic signature of lung adenocarcinoma.

Previous studies have investigated the prognostic role of immune-related genes (IRGs) in lung adenocarcinoma from the ImmPort database (10, 11); however, this database contains published data on IRGs, thus potentially not accounting for all IRGs. Moreover, these studies have reported no correlation between prognostic factors and OS among certain subgroups of patients with lung adenocarcinoma, indicating that this association is largely unknown. One of the important reasons may be the complex prognostic behavior of tumors; furthermore, when considering the characteristics of IRGs directly associated with tumors, it is also important to focus on the tumor microenvironment (TME) (12). The TME is closely associated with tumorigenesis and patient prognosis (13, 14). Moreover, accumulating evidence indicates that tumor-infiltrating immune cells and stromal cells (15, 16), as the primary nontumor components of the TME, play a significant role in lung cancer prognosis. These findings highlight the importance of understanding the association between the TME and lung adenocarcinoma prognosis. The development of a prognostic model of IRGs based on the TME might provide novel insights into the generation of a more accurate prognostic system.

Accurate management and appropriate personalized therapies for lung adenocarcinoma are required in accordance with prognostic stratification. Moreover, an enhanced understanding of IRGs involved in the TME would help elucidate their regulatory mechanisms in the TME and develop new treatment strategies. With advancements in machine learning, the ESTIMATE algorithm has been used to investigate IRGs in the TME, based on the immune and stromal scores of the TME, and to generate a TME-related immune prognostic model (17). Moreover, this algorithm can effectively predict the prognosis of patients with various cancers. Accordingly, in this study, we determined the immune and stromal scores of tumors using the ESTIMATE algorithm and developed a novel TME-related immune prognostic model of lung adenocarcinoma.

## Materials and Methods

### Acquisition of TCGA Data

Normalization of RNA sequence data, in terms of level 3 fragments per kilobase of exon per million fragments mapped (FPKM) reads, was performed for 594 samples obtained from The Cancer Genome Atlas (TCGA) database, including 535 adenocarcinoma and 59 normal lung samples, before December 15, 2019. Thereafter, the Ensemble IDs were converted to gene symbols in accordance with human gene annotations. Furthermore, clinical data of lung adenocarcinoma patients were obtained and merged into a single matrix for subsequent analysis. Patients with an incomplete follow-up duration or recorded date of death of any cause were excluded. Finally, 494 lung adenocarcinoma patients with expression profiles and clinical data were included.

### Immune Score and Stromal Score in the TME

Estimation of STromal and Immune cells in MAlignant Tumor tissues using Expression data (ESTIMATE) is a tool for predicting and estimating infiltrating immune and stromal cells in tumor tissues based on gene expression profiles. Herein, the ESTIMATE algorithm was used to analyze the characteristics of specific gene expression in immune and stromal cells for each tumor sample to predict their immune and stromal scores. Thereafter, the immune and stromal scores were analyzed using the estimate package in R software.

### Screening of Differentially Expressed IRGs

Based on the median immune and stromal scores, patients with lung adenocarcinoma were divided into two groups: high and low immune/stromal score groups. Significant differences in OS between the high and low immune/stromal score groups were analyzed. On the basis of the significant differences in patient prognosis, differentially expressed IRGs between the two groups were assessed using the Limma package in R software. Finally, intersecting differentially expressed IRGs in both groups were considered for further analysis. A log(fold change) of >2 and an adjusted *p*-value of <0.05 were considered cutoffs. Heat maps and Venn diagrams were generated using R.

### GO and KEGG Pathway Enrichment Analyses of Differentially Expressed IRGs

Gene Ontology (GO) and Kyoto Encyclopedia of Genes and Genomes (KEGG) pathway enrichment analyses were performed to understand gene functional annotation and functional enrichment, respectively. Common differentially expressed IRGs of GO and KEGG annotation were performed using Database for Annotation, Visualization, and Integrated Discovery (DAVID), an online database (https://david.ncifcrf.gov/), to predict functional domains and their biological implications. Fisher's exact test was performed to analyze pathways, diseases, and functions. A *p*-value of <0.05 indicated the significance of GO terms and KEGG pathway enrichment in genes herein. Furthermore, the top 10 GO terms and KEGG pathway enrichment results were mapped using Hmisc and ggplot2 in R software.

### Construction of Prognostic Model for Lung Adenocarcinoma in the Training Set

A total of 494 patients with lung adenocarcinoma in the TCGA dataset were randomly divided into a training set and testing set at a ratio of 7:3 (training cohort, 346 patients; testing cohort, 148 patients).

First, univariate Cox proportional hazard regression analysis was performed to screen out prognostic IRGs in the training cohort (iteration = 1,000), with *p* < 0.05 indicating statistical significance. Second, the key IRGs were further selected from among significant prognostic IRGs on univariate analysis, through least absolute shrinkage and selection operator (LASSO) regression, a powerful tool for developing refined prognostic models, fitting generalized linear models, selecting variables, and regularizing complexity, using R software. Key IRGs were subjected to multivariate Cox regression analysis. Finally, the risk score formula was developed in accordance with the key IRGs identified through LASSO analysis.

### Evaluation of the Prognostic Model in the Training Set

After the expression value of each specific gene was included, the risk score formula for each patient was weighted by its estimated regression coefficient on LASSO regression analysis. On the basis of the best separation of risk score, patients were divided into high-risk-score and low-risk-score groups. Survival differences between the two groups were assessed by Kaplan–Meier survival curves using log-rank tests. ROC curves were used to assess the accuracy of model prediction. Furthermore, LASSO regression analysis was performed to examine the role of the risk score in predicting clinical outcomes. Furthermore, the association between risk score and clinical stage was analyzed.

To further determine whether the independent prognostic model could be used as an independent prognostic factor, univariate and multivariate Cox regression analyses were performed to analyze the predictive value of age, sex, stage, TNM stage, and predictive model. In the univariate analysis, the correlation between some independent variables and dependent variables was considered, and some correlations might be masked by the influence of confounding factors. To avoid omitting important predictors of prognosis, the threshold in univariate analysis was relaxed to *p* < 0.1, while the *p*-value in multivariate analysis was still 0.05.

### GSEA Analysis of Differences in Pathway Enrichment in the Training Set

To investigate the differences in the putative mechanism between the high-risk-score and low-risk-score groups, gene set enrichment analysis (GSEA) was performed to comprehensively analyze the differences in function enrichment. GSEA is a computational method of determining whether an a priori defined set of genes is significantly different between two biological states. The number of permutations was set to 1,000, and the permutation type was set to phenotype. In this study, all genes in the training set were sequenced according to the degree of differential expression in the high-risk-score group and the low-risk-score group. GSEA was used to comprehensively analyze differences in gene pathway enrichment between the two groups.

### Validation of the Prognostic Model in the Testing Cohort

The prognostic model was further validated for the testing cohort (*n* = 148).

Similarly, the risk score of each patient was weighted on the basis of the risk score. Thereafter, based on the best separation of the risk score, patients in the testing cohort were divided into high-risk-score and low-risk-score groups. A Kaplan–Meier survival curve and ROC curve analysis were performed in the testing however.

### Functional Analysis of IRG Signatures in the Model

To further analyze the mutation characteristics and the putative functional mechanisms of these hub IRGs in lung adenocarcinoma, gene expression profiles of patients with lung adenocarcinoma were imported from the following datasets: TCGA database (Broad, Cell 2012), Lung Adenocarcinoma (MSKCC, Science 2015), Lung Adenocarcinoma (TCGA, Firehose Legacy), Lung Adenocarcinoma (TSP, Nature 2008), and Non-Small-Cell Cancer (MSKCC, Cancer Discov 2017). A combined study of five datasets including 1,825 patients were included in this study.

GSCALite is an online cancer genomic analysis tool that integrates cancer genomics data for 33 cancer types from the TCGA and normal tissue data from GTEx (http://bioinfo.life.hust.edu.cn/web/GSCALite/), enabling gene set pathway analysis in data analysis. In this study, GSCALite was used to analyze the pathway of hub genes.

The TIMER database is a comprehensive tool for analyzing the immune cell infiltrates in tumors. The abundances of six immune infiltrates (B cells, CD4+ T cells, CD8+ T cells, neutrophils, macrophages, and dendritic cells) were estimated using the TIMER algorithm. In this study, the TIMER database was further applied to analyze the correlation between hub genes and immune cells. The correlation of hub gene expression with immune infiltration level was visualized in lung adenocarcinoma using the Gene module. The scatterplots were generated and displayed after hub genes and cancer type were submitted successfully, showing the purity-corrected partial Spearman's correlation and statistical significance.

### Statistical Analysis

All statistical analyses were conducted with the R language (version 3.6.1). All statistical tests were bilateral, and *p* < 0.05 was statistically significant.

## Results

### Study Design and Workflow Overview

**Figure 1:**
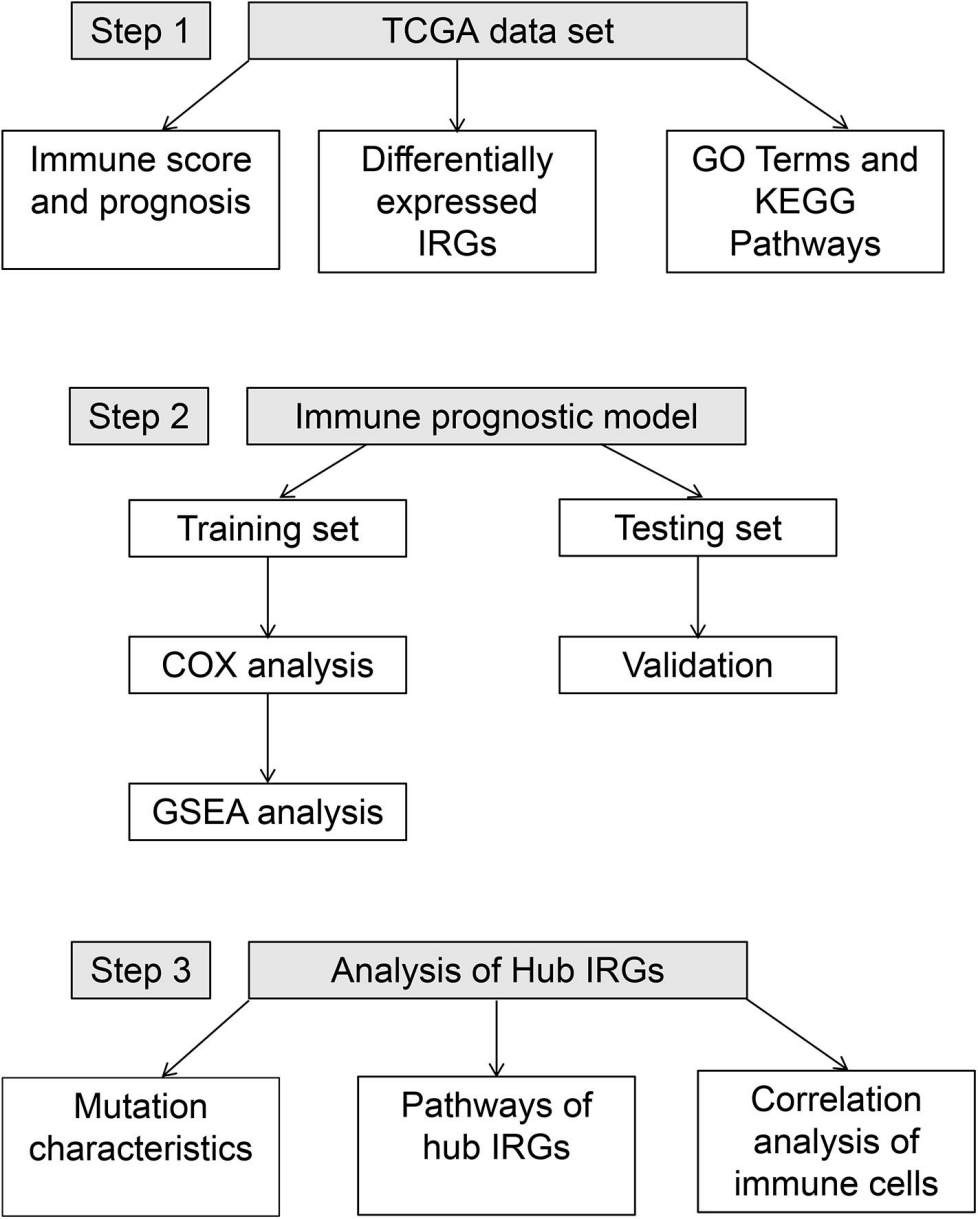
Workflow of the construction and verification of the immune-stromal score prognostic signature.

A total of 594 samples were obtained, including 535 adenocarcinoma and 59 normal lung samples. Data for a total of 494 lung adenocarcinoma patients with clinical information were retrieved (Table 1). The workflow for constructing and verifying the immune-related prognostic model is shown in Figure 1.

**Table 1:** Baseline characteristics of patients with lung adenocarcinoma.

	**Training set**		**Testing set**		***p*-value**
	**High score** **(*N* = 194)**	**Low score** **(*N* = 152)**	**High score** **(*N* = 91)**	**Low score** **(*N* = 57)**	
**Age (years)**					
≥60	132	116	68	42	0.131
<60	62	36	23	15	0.767
**Gender**					
Male	92	67	55	14	0.002
Female	102	85	36	42	0.213
**AJCC stage**					
Stage I	95	99	35	34	0.802
Stage II	47	29	28	13	0.488
Stage III	41	17	18	4	0.312
Stage IV	9	4	9	4	1.000
NA	2	3	1	2	0.850
**T stage**					
T1	53	70	22	22	0.429
T2	108	68	52	28	0.577
T3	22	9	11	3	0.593
T4	9	5	5	0	0.257
TX	2	0	1	0	NA
**N stage**					
N0	114	115	50	39	0.305
N1–3	74	34	40	16	0.701
NX	6	3	1	2	0.523
**M stage**					
M0	133	92	60	40	0.880
M1	8	4	9	4	1.000
MX	51	54	23	13	0.112
NA	2	2	0	0	NA
Survival Time (days)	653.45 ± 36.96	840.44 ± 82.79	714.41 ± 95.64	953.81 ± 118.03	NA
**Survival status**					
Alive	113	124	51	44	0.323
Dead	81	28	40	13	0.873

### Immune Score and Stromal Score in the TME

**Figure 2:**
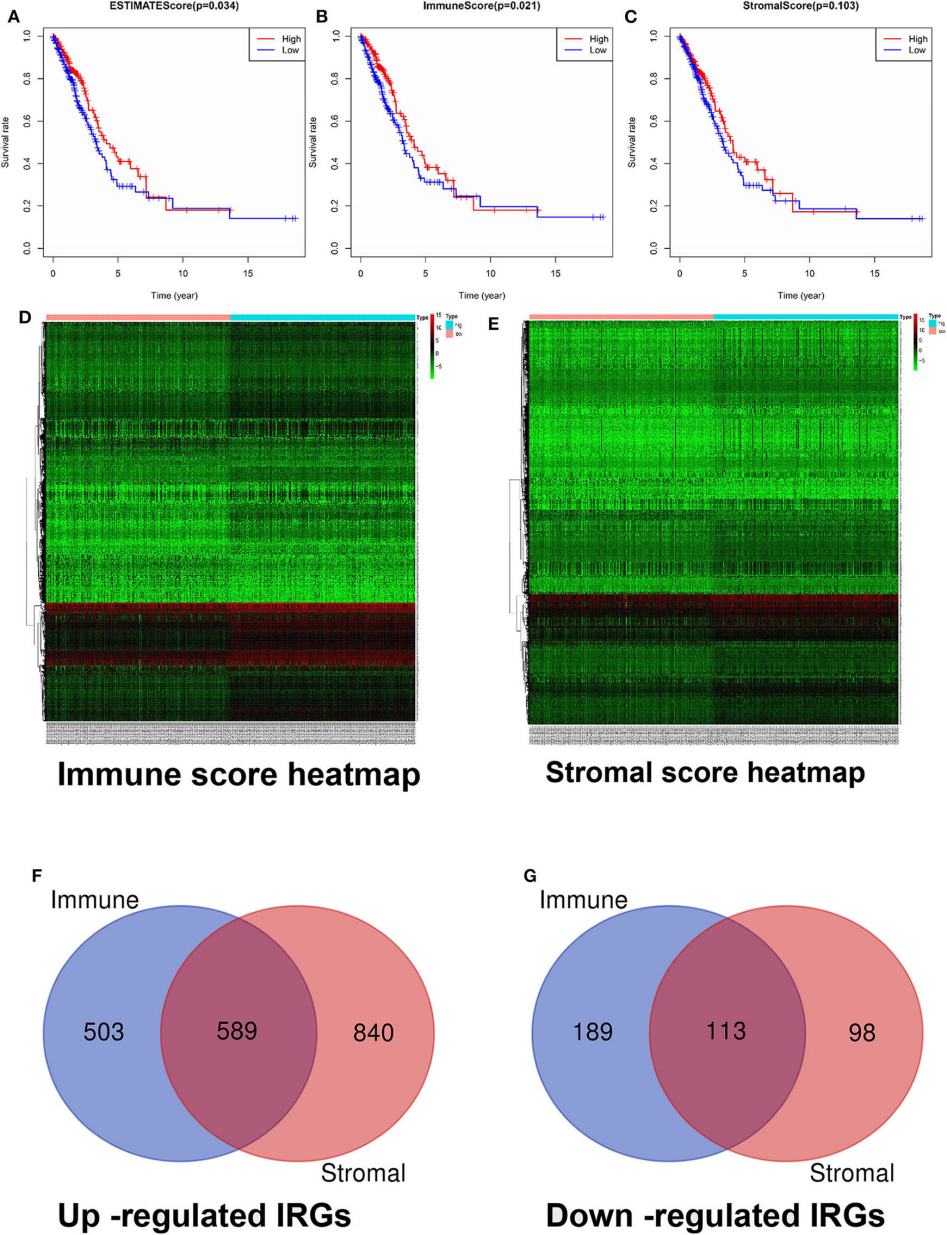
Screening and identification of differentially expressed IRGs. **(A)** Relationship between comprehensive immune score and OS. **(B)** Relationship between immune score and OS. **(C)** Relationship between stromal score and OS. **(D)** Heatmap of immune score groups. **(E)** Heatmap of stromal score groups. **(F)** The Venn diagram of the intersection of up-regulated IRGs between the immune and stromal score groups. **(G)** The Venn diagram of the intersection of down-regulated IRGs between the immune and stromal score groups. OS, overall survival; IRGs, immune-related genes.

The immune and stromal scores were analyzed using the ESTIMATE algorithm. The immune and stromal scores of lung adenocarcinoma are shown in Supplementary Table 1. On the basis of the median value of immune and stromal scores, patients with lung adenocarcinoma were divided into two groups: the high immune/stromal score group and the low score group. These results show that the high immune score group had better OS than the low immune score group for lung adenocarcinoma. In terms of stromal score, although patients with high stromal score had better prognosis than those with low stromal score, the difference was not statistically significant (Figures 2A–C).

### Screening of Differentially Expressed IRGs

According to the criteria of a log(fold change) of >2 and an adjusted *p*-value of <0.05, our results showed that a total of 3,034 genes with significant differentially expressed IRGs were screened, including 2,521 upregulated IRGs and 513 downregulated IRGs. Among them, 1,394 differentially expressed IRGs (1,092 upregulated IRGs and 302 downregulated IRGs) were included in the immune score group (Supplementary Table 2), and 1,640 differentially expressed IRGs (1,429 upregulated IRGs and 211 downregulated IRGs) were included in the stromal score group (Supplementary Table 3). Heat maps and Venn diagrams are displayed in Figures 2D–G. In total, 702 intersecting differentially expressed IRGs (589 upregulated and 113 downregulated) in both groups are indicated in Figures 2F,G (Supplementary Table 4).

### GO Terms and KEGG Pathway Enrichment Analysis of Differentially Expressed IRGs

**Figure 3:**
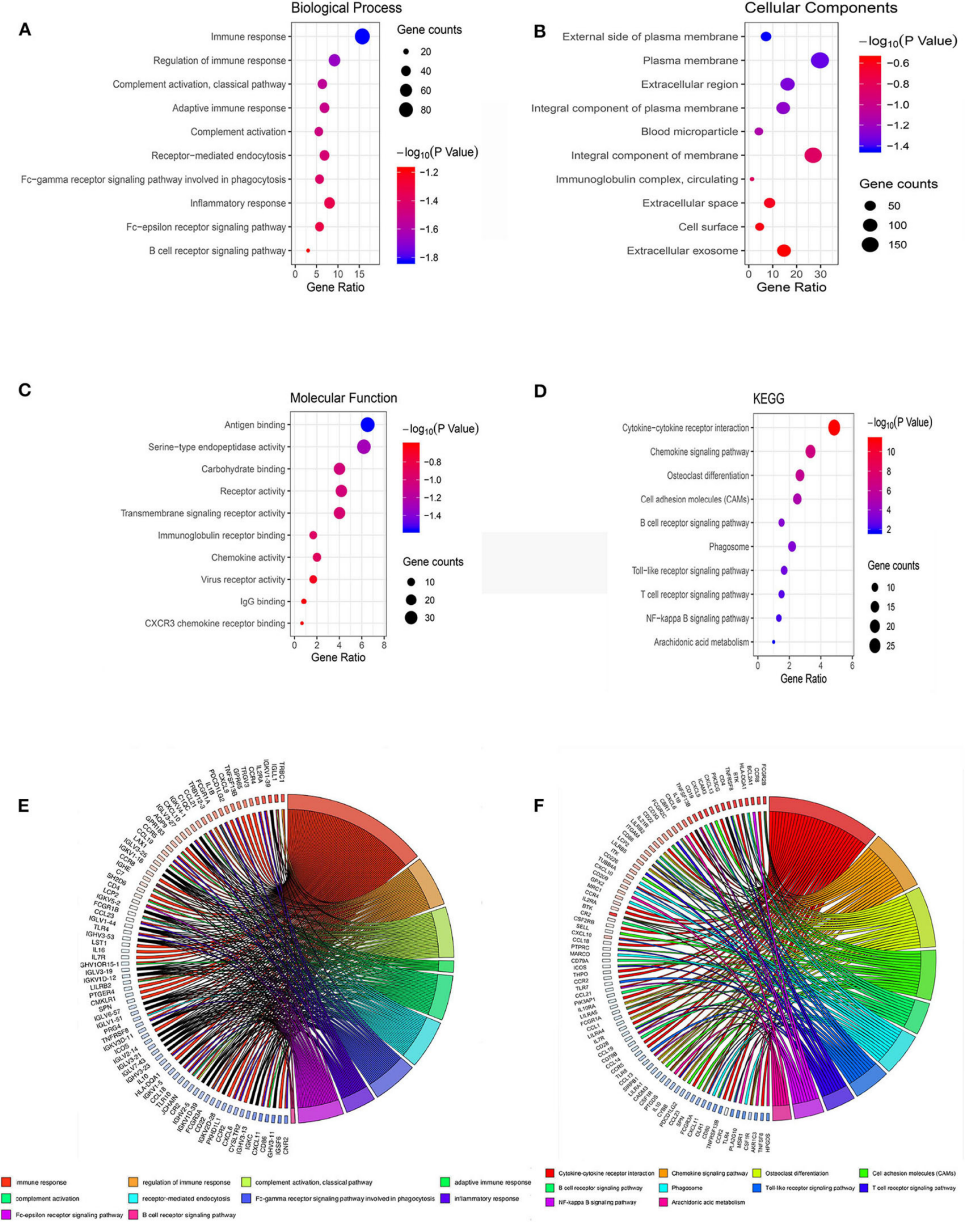
Bubble map of the top 10 GO terms and KEGG pathway enrichment analysis data of differentially expressed IRGs. **(A)** GO analysis of differentially expressed IRGs in biological processes. **(B)** GO analysis of differentially expressed IRGs in cellular components. **(C)** GO analysis of differentially expressed IRGs in terms of molecular function. **(D)** KEGG enrichment analysis of differentially expressed IRGs. A high gene ratio represents a high level of enrichment. The size of the dot indicates the number of target genes in the pathway, and the color of the dot reflects the *p*-value range. **(E)** GO chord plot of differentially expressed IRGs. **(F)** KEGG chord plot of differentially expressed IRGs.

GO terms are divided into three parts: biological processes, cellular components, and molecular functions. GO analysis showed that upregulated IRGs were mainly involved in the immune response (BP, GO: 0006955), external side of plasma membrane (CC, GO: 0009897), and antigen binding (MF, GO: 0003823). The downregulated IRGs were mainly involved in the cellular response to jasmonic acid stimulus (BP, GO: 0071395), neuronal cell body (CC, GO: 0043025), and oxidoreductase activity, and in acting on NAD(P)H, quinone or similar compounds as acceptors (MF, GO: 0016655) (Figures 3A–C,E; Table 2). The KEGG pathway enrichment results in the upregulated IRGs were mainly involved in cytokine–cytokine receptor interactions, chemokine signaling pathways, and cell adhesion molecules (CAMs). However, the KEGG pathway enrichment results of downregulated IRGs were mainly involved in arachidonic acid metabolism, metabolic pathways, and tyrosine metabolism (Figures 3D,F; Table 3).

**Table 2:** Top five GO terms.

**Category**	**ID**	**Term**	**Count**	**%**	***p* value**	**FDR**
**Upregulated IRGs**						
GOTERM_BP_DIRECT	GO:0006955	Immune response	93	17.95366795	4.59E−75	7.47E−72
GOTERM_BP_DIRECT	GO:0050776	Regulation of immune response	55	10.61776062	1.14E−51	1.86E−48
GOTERM_BP_DIRECT	GO:0002250	Adaptive immune response	41	7.915057915	4.73E−36	7.69E−33
GOTERM_BP_DIRECT	GO:0006956	Complement activation	33	6.370656371	6.95E−34	1.13E−30
GOTERM_BP_DIRECT	GO:0006898	Receptor-mediated endocytosis	40	7.722007722	1.48E−30	2.40E−27
GOTERM_CC_DIRECT	GO:0009897	External side of plasma membrane	43	8.301158301	1.52E−31	1.86E−28
GOTERM_CC_DIRECT	GO:0005886	Plasma membrane	167	32.23938224	5.95E−29	7.29E−26
GOTERM_CC_DIRECT	GO:0005887	Integral component of plasma membrane	85	16.40926641	2.95E−23	3.61E−20
GOTERM_CC_DIRECT	GO:0005576	Extracellular region	86	16.6023166	3.11E−20	3.81E−17
GOTERM_CC_DIRECT	GO:0072562	Blood microparticle	24	4.633204633	4.61E−15	5.71E−12
GOTERM_MF_DIRECT	GO:0003823	Antigen binding	39	7.528957529	6.92E−41	9.44E−38
GOTERM_MF_DIRECT	GO:0004252	Serine-type endopeptidase activity	36	6.94980695	1.11E−21	1.52E−18
GOTERM_MF_DIRECT	GO:0004872	Receptor activity	25	4.826254826	4.79E−13	6.53E−10
GOTERM_MF_DIRECT	GO:0004888	Transmembrane signaling receptor activity	24	4.633204633	2.73E−12	3.72E−09
GOTERM_MF_DIRECT	GO:0030246	Carbohydrate binding	23	4.44015444	3.43E−12	4.68E−09
**Downregulated IRGs**						
GOTERM_BP_DIRECT	GO:0071395	Cellular response to jasmonic acid stimulus	3	3.75	7.50E−05	0.106090023
GOTERM_BP_DIRECT	GO:0044598	Doxorubicin metabolic process	3	3.75	3.47E−04	0.489670004
GOTERM_BP_DIRECT	GO:0044597	Daunorubicin metabolic process	3	3.75	3.47E−04	0.489670004
GOTERM_BP_DIRECT	GO:0030855	Epithelial cell differentiation	4	5	0.002002754	2.797891239
GOTERM_BP_DIRECT	GO:0055114	Oxidation–reduction process	8	10	0.004999641	6.849000785
GOTERM_CC_DIRECT	GO:0043025	Neuronal cell body	6	7.5	0.00495641	5.157580931
GOTERM_CC_DIRECT	GO:0008076	Voltage-gated potassium channel complex	3	3.75	0.038309612	34.05203615
GOTERM_CC_DIRECT	GO:0005576	Extracellular region	11	13.75	0.053136406	44.11602637
GOTERM_CC_DIRECT	GO:0005892	Acetylcholine-gated channel complex	2	2.5	0.081015685	59.35918267
GOTERM_MF_DIRECT	GO:0016655	Oxidoreductase activity, acting on NAD(P)H, quinone or similar compound as acceptor	4	5	2.36E−06	0.002915475
GOTERM_MF_DIRECT	GO:0047086	Ketosteroid monooxygenase activity	3	3.75	3.72E−05	0.045918231
GOTERM_MF_DIRECT	GO:0047115	trans-1,2-dihydrobenzene-1,2-diol dehydrogenase activity	3	3.75	7.42E−05	0.091606887
GOTERM_MF_DIRECT	GO:0018636	Phenanthrene 9,10-monooxygenase activity	3	3.75	7.42E−05	0.091606887
GOTERM_MF_DIRECT	GO:0004032	Alditol:NADP+ 1-oxidoreductase activity	3	3.75	2.58E−04	0.318095483

**Table 3:** Top five KEGG pathway enrichment results.

**Category**	**Term**	**Count**	**%**	***p*-value**	**Genes**	**FDR**
**Upregulated IRGs**						
KEGG_PATHWAY	Cytokine–cytokine receptor interaction	28	5.405405405	2.94E−13	CCL1, IL21R, CXCL9, TNFRSF8, CXCL6, CXCL11, IL7R, IL10, CXCL10, CCL23, CCL21, IL10RA, CSF2RB, IL1B, CSF1R, IL2RA, TNFRSF13B, CCL19, CCL18, TNFSF8, CCR8, CCL13, CCL14, TNFSF13B, CCR5, CCR4, CXCL13, CCR2	3.53E−10
KEGG_PATHWAY	Chemokine signaling pathway	19	3.667953668	3.51E−08	CCL1, PIK3CG, ITK, CXCL9, CCL19, CXCL6, CXCL11, CCL18, CXCL10, CCR8, DOCK2, CCL13, CCL23, CCL14, CCR5, CCL21, CXCL13, CCR4, CCR2	4.22E−05
KEGG_PATHWAY	Cell adhesion molecules (CAMs)	15	2.895752896	1.03E−06	PTPRC, CADM3, SELL, ICAM3, ITGAM, PDCD1LG2, HLA-DQA1, CD86, CD80, ICOS, CD22, CD4, CD226, SPN, CD28	0.00123743
KEGG_PATHWAY	B cell receptor signaling pathway	9	1.737451737	7.44E−05	PIK3CG, CD19, CR2, FCGR2B, CD22, PIK3AP1, CD79B, CD79A, BTK	0.089383945
KEGG_PATHWAY	Phagosome	12	2.316602317	2.37E−04	MRC1, MARCO, MSR1, FCGR2B, OLR1, FCGR2C, FCGR1A, CD209, TLR4, FCGR3A, ITGAM, HLA-DQA1	0.284714595
**Downregulated IRGs**						
KEGG_PATHWAY	Arachidonic acid metabolism	4	5	0.002057483	AKR1C3, GPX2, CBR1, PLA2G10	2.102810979
KEGG_PATHWAY	Metabolic pathways	13	16.25	0.002420062	ETNPPL, DDC, ODC1, PLA2G10, OGDHL, HAL, HGD, TAT, AKR1C3, CBR1, HMGCS2, ENO3, NAT8L	2.469211516
KEGG_PATHWAY	Tyrosine metabolism	3	3.75	0.009368241	DDC, HGD, TAT	9.255598588
KEGG_PATHWAY	Metabolism of xenobiotics by cytochrome P450	3	3.75	0.038441569	AKR1C2, CBR1, AKR1C1	33.26837318
KEGG_PATHWAY	Phenylalanine metabolism	2	2.5	0.069378576	DDC, TAT	52.38108893

### Development of a Prognostic Model for the Training Cohort

To generate a prognostic model for lung adenocarcinoma, univariate regression analysis was first performed to screen the key prognostic genes in the training cohort. Thereafter, 58 significantly differentially expressed IRGs correlated with prognosis were considered for LASSO regression analysis. Finally, three key IRGs (*CLEC17A, INHA*, and *XIRP1*) were selected to generate an immune prognostic model. The results of the multivariate Cox regression analysis are summarized in Table 4. The risk score was determined using the following formula:

**Table 4:** Multivariate Cox regression analysis of key immune-related genes.

**Gene**	**Coef**	**HR**	**HR.95L**	**HR.95H**	***p*-value**
CLEC17A	−0.13549042	0.192289385	0.071601478	0.516402855	0.001071204
INHA	0.01207179	1.141665832	1.046649718	1.245307623	0.002804616
XIRP1	0.6263501	2.024671234	1.219239069	3.362173761	0.006410729

[Expression level of *CLEC17A* × (−0.13549042)] + [Expression level of *INHA* × (0.01207179)] + [Expression level of *XIRP1* × (0.6263501)].

### Evaluation of the Prognostic Model in the Training Cohort

**Figure 4:**
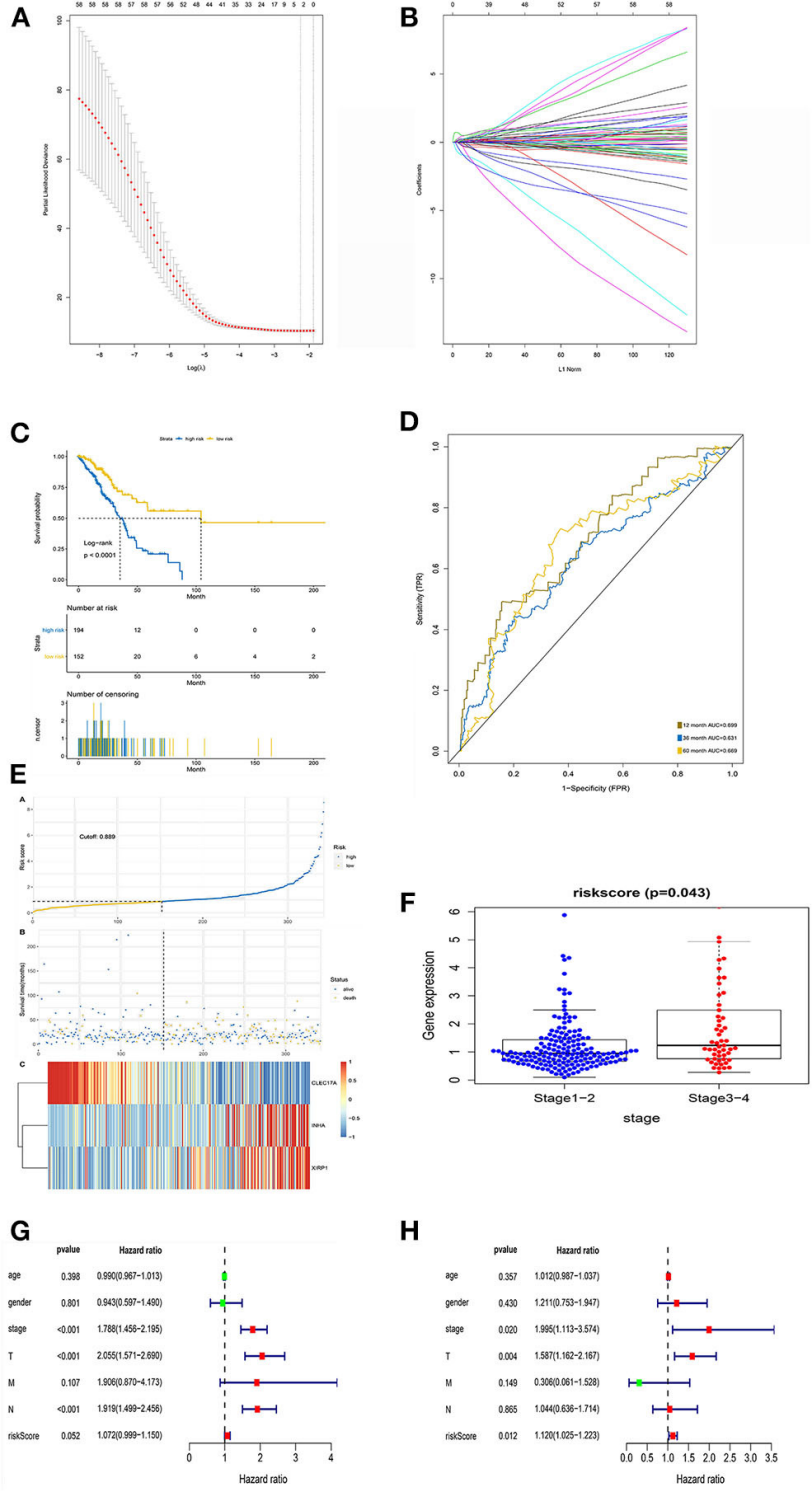
LASSO regression analysis and identification of prognostic signatures in the training set. **(A)** Ten-fold cross-validation for turning parameter selection in the LASSO Cox regression model. **(B)** Coefficient profiles in the LASSO Cox regression model. **(C)** Survival curve of low- and high-risk groups stratified by immune-stromal score signature. **(D)** ROC analysis of the TCGA dataset for prognostic signature. **(E)** LASSO regression analysis of low- and high-risk groups. **(F)** Relationship between risk score and clinical stage. **(G)** The forest plot of the prognostic signature by univariate analysis. **(H)** The forest plot of the prognostic signature by multivariate Cox proportional regression analysis.

LASSO regression analysis was performed to construct and evaluate the prognostic model (Figures 4A,B). Patients were divided into high- and low-risk-score groups in accordance with the best separation of risk scores. The high-risk-score group had significantly worse OS than the low-risk-score group (*p* < 0.0001; Figure 4C). The area under the ROC curve for predicting the 1-, 3-, and 5-year survival of lung adenocarcinoma was 0.699, 0.631, and 0.669, respectively (Figure 4D).

Additionally, the risk curve indicated that the high-risk-score group had a higher mortality and worse prognosis than the low-risk-score group (cutoff value: 0.889; Figure 4E). Further analysis of the relationship between risk score and pathological stage revealed that patients with early-stage lung adenocarcinoma (stages 1 and 2) scored lower than those with advanced stage lung adenocarcinoma (*p* = 0.043; Figure 4F).

Univariate Cox analysis showed that pathological staging and risk score had statistical significance, while age and sex had no statistical significance (Figure 4G). However, multivariate Cox analysis showed that pathological stage (HR, 1.995; 95% CI, 1.113–3.574; *p* = 0.020) and risk score (HR, 1.120; 95% CI, 1.025–1.223; *p* = 0.012) were independent prognostic factors (Figure 4H).

### GSEA of the Mechanism Underlying the Prognostic Differences Between the Two Groups

**Figure 5:**
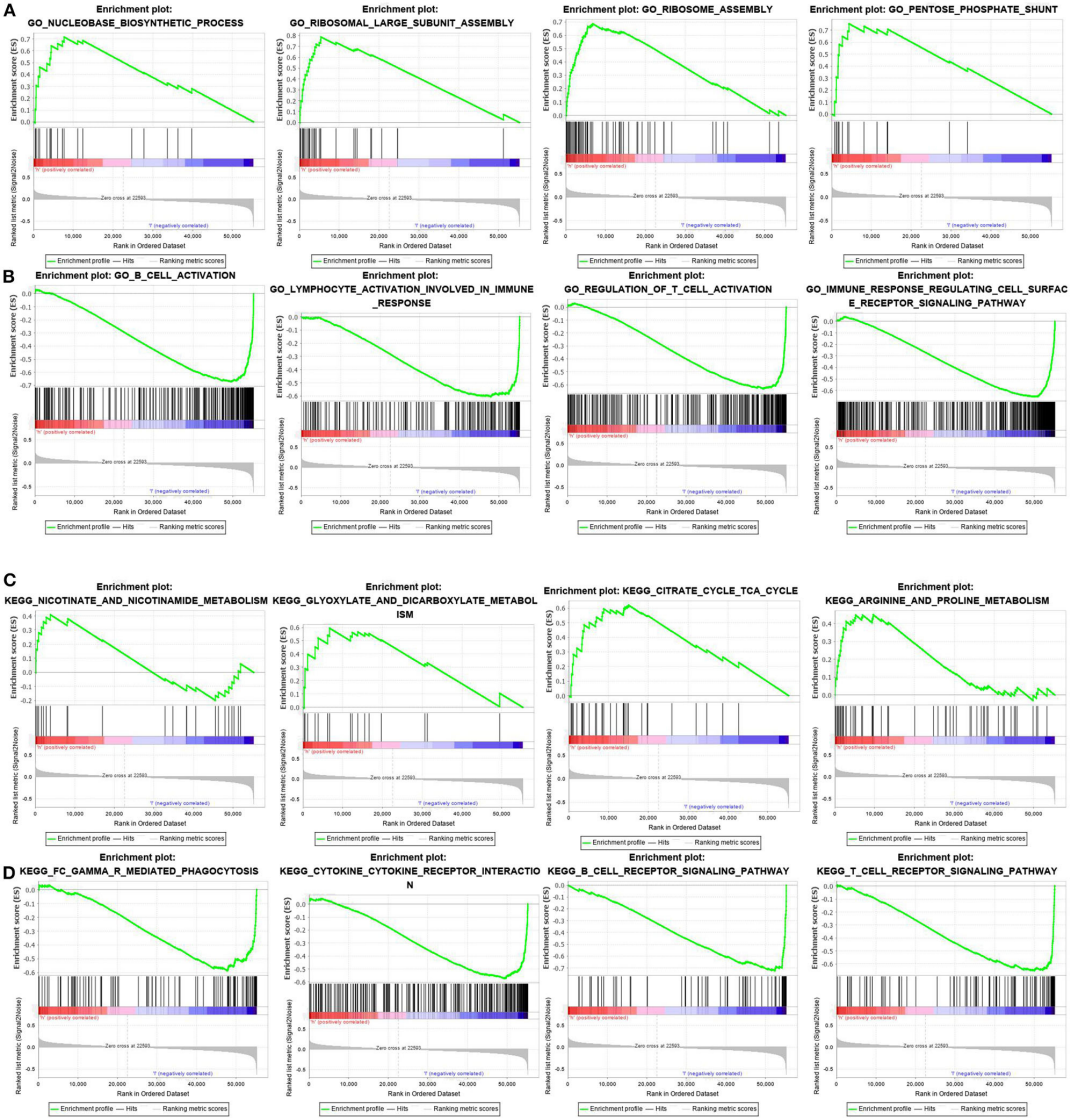
GSEA analysis of differences in pathway enrichment in the training set. **(A,B)** GO terms in the high- and low-risk score groups. **(C,D)** KEGG pathway enrichment in the high- and low-risk score groups.

In this study, the possible molecular mechanisms of the prognosis difference between the two groups of patients were analyzed by GSEA analysis. The results showed that the GO and pathway enrichment in the high-risk-score group was mainly involved in metabolism-related pathways (Figures 5A,C). However, GO and pathway enrichment in the low-risk-score group was primarily focused on immunoregulation and immune cell activation (Figures 5B,D). The detailed GSEA results are described in Table 5.

**Table 5:** Detailed results of gene set enrichment analysis.

**NAME**	**ES**	**NES**	**NOM *p* value**
**GO Terms**			
**High score group**			
GO_RIBOSOMAL_LARGE_SUBUNIT_ASSEMBLY	0.78643316	1.9444792	0.00203252
GO_PENTOSE_PHOSPHATE_SHUNT	0.7535411	1.8539398	0.008298756
GO_RIBOSOME_ASSEMBLY	0.68597955	1.8540714	0.016528925
GO_NUCLEOBASE_BIOSYNTHETIC_PROCESS	0.71662575	1.7974799	0.01980198
**Low score group**			
GO_B_CELL_ACTIVATION	−0.6719656	−2.3789294	0
GO_IMMUNE_RESPONSE_REGULATING_CELL_SURFACE_RECEPTOR_SIGNALING_PATHWAY	−0.654903	−2.3589978	0
GO_LYMPHOCYTE_ACTIVATION_INVOLVED_IN_IMMUNE_RESPONSE	−0.60444784	−2.3353372	0
GO_T_CELL_ACTIVATION	−0.6102059	−2.343195	0
**KEGG Pathway Enrichment**			
**High score group**			
KEGG_CITRATE_CYCLE_TCA_CYCLE	0.62047356	1.6142783	0.046184737
KEGG_GLYOXYLATE_AND_DICARBOXYLATE_METABOLISM	0.5940425	1.5761957	0.060194176
KEGG_ARGININE_AND_PROLINE_METABOLISM	0.45005503	1.4639851	0.055900622
KEGG_NICOTINATE_AND_NICOTINAMIDE_METABOLISM	0.4092993	1.3358487	0.115384616
**Low score group**			
KEGG_B_CELL_RECEPTOR_SIGNALING_PATHWAY	−0.72458243	−2.4734645	0
KEGG_T_CELL_RECEPTOR_SIGNALING_PATHWAY	−0.6531744	−2.3155243	0
KEGG_CYTOKINE_CYTOKINE_RECEPTOR_INTERACTION	−0.5735697	−2.178205	0.001968504
KEGG_FC_GAMMA_R_MEDIATED_PHAGOCYTOSIS	−0.5871511	−2.1173847	0

### Validation of the Prediction Model in the Testing Cohort

**Figure 6:**
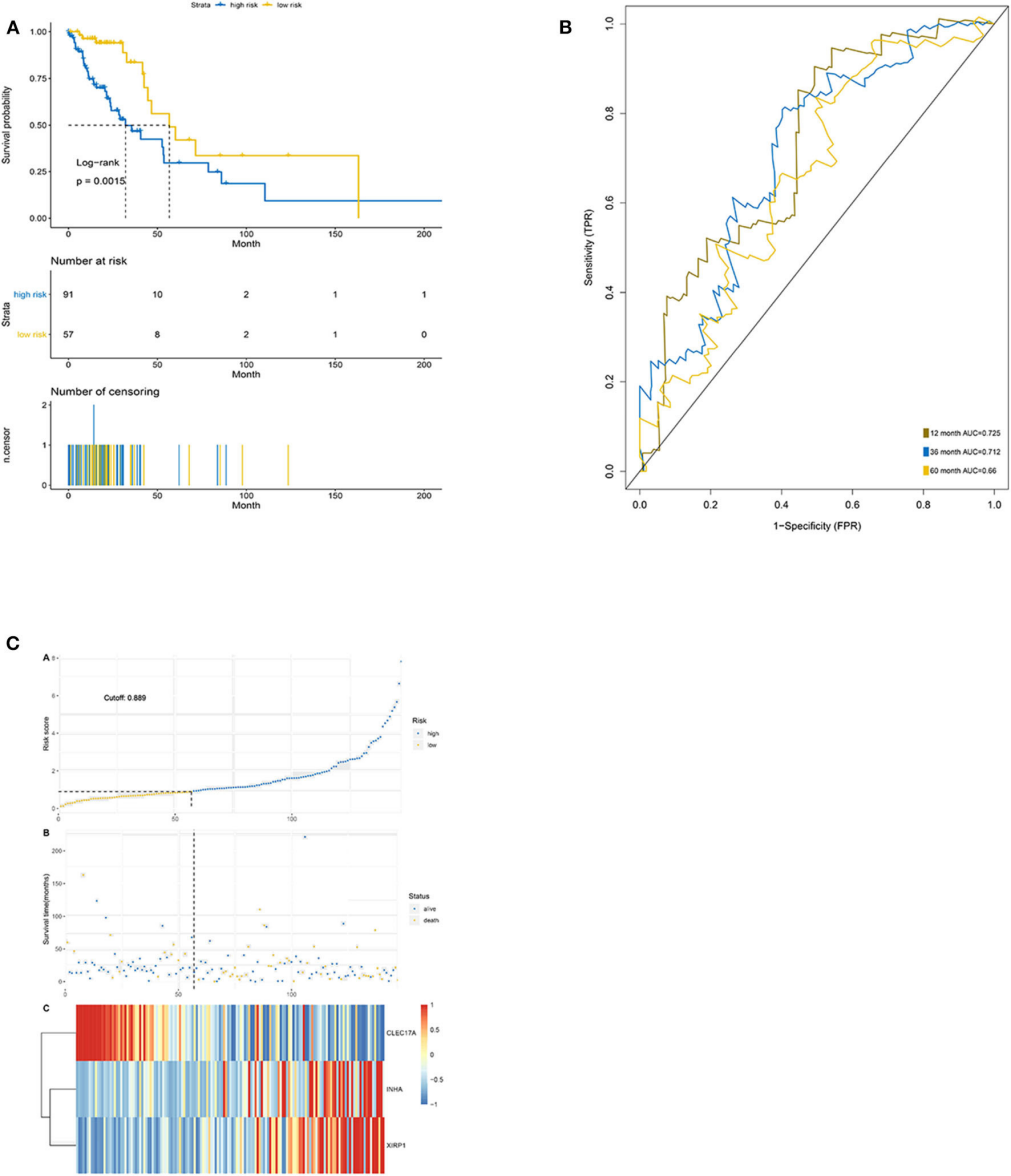
Validation of the prediction features. **(A)** Survival curve of low- and high-risk groups stratified by immune-stromal score signature. **(B)** ROC analysis of the TCGA dataset for prognostic signature. **(C)** LASSO regression analysis of low- and high-risk groups.

The Kaplan–Meier results showed that the high-risk group had worse OS than the low-risk group (*p* < 0.0001) (Figure 6A). The area under the ROC curve for predicting the 1-, 3-, and 5-year survival of lung adenocarcinoma was 0.725, 0.712, and 0.660, respectively (Figure 6B). Additionally, risk curve revealed that the high-risk-score group had a worse prognosis than the low-risk-score group (Figure 6C). These results were consistent with the results of the training set.

### The Mechanism of Action of Hub IRGs in the TCGA Database

**Figure 7:**
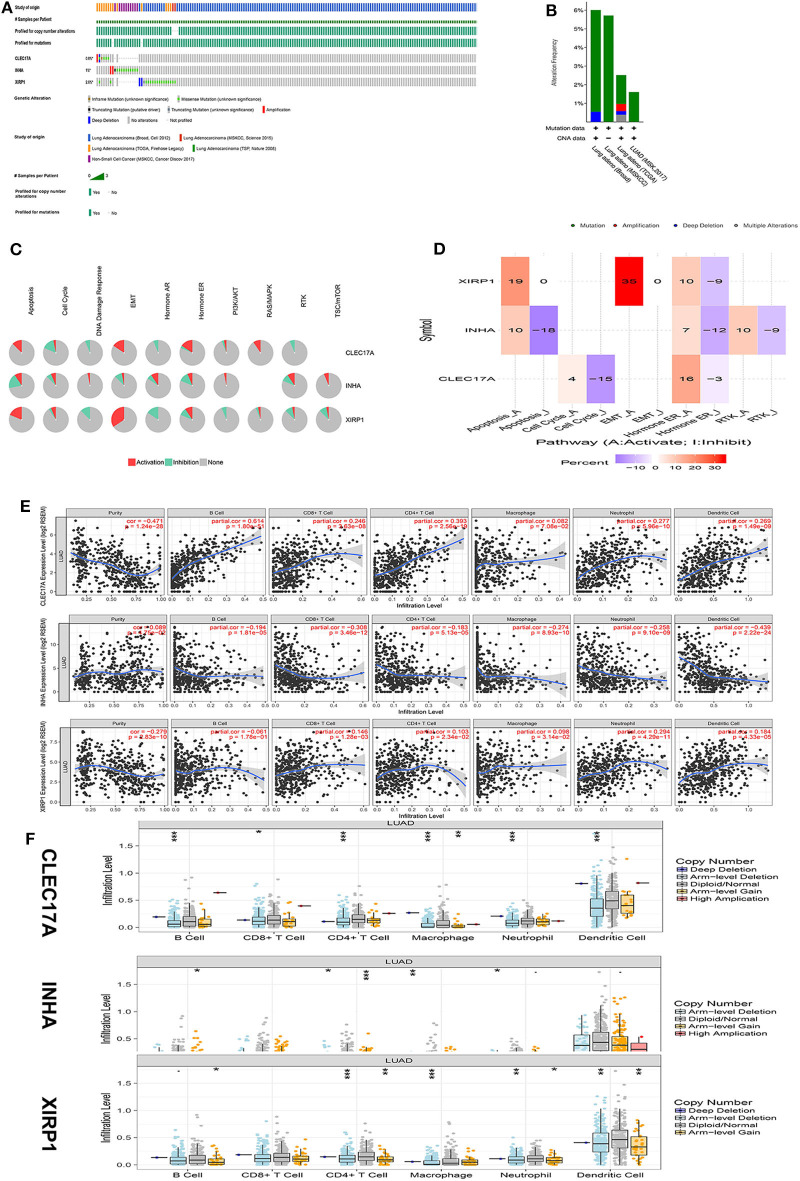
Mechanism analysis of hub genes in the TCGA database. **(A)** Matrix heat map shows genomic alterations of hub genes in five lung datasets. **(B)** The alteration frequencies of hub genes across five studies on lung adenocarcinoma. **(C)** The pathways of hub genes were analyzed by the GSCALite tool. **(D)** Heatmap percentage of hub genes. **(E)** The correlation between the hub gene and immune cells was analyzed in the TIMER database. **(F)** The relationship between copy number variation (CNV) of hub genes and immune cell infiltration was further analyzed.

To further analyze the potential function of hub IRGs, our results were verified using the TCGA and GTEx databases. First, the mutation characteristics of these hub IRGs were analyzed among patients with lung adenocarcinoma. The mutation rates of these hub IRGs in patients with lung adenocarcinoma were 0.8, 1, and 2.6% (Figure 7A). Moreover, each hub gene had different mutation forms, including mutation, deletion, and amplification, in lung adenocarcinoma. For example, the mutation form of CLEC17A was mainly amplification, the mutation form of INHA was mainly amplification and missense mutation, while the mutation form of XIRP1 was mainly deep deletion and missense mutation (Figure 7B).

In addition, analysis of pathways in the GSCALite database revealed that CLEC17A is primarily involved in the activation of the epithelial–mesenchymal transition (EMT) and RAS pathways and cell cycle inhibition. INHA is primarily involved in the activation of the mTOR pathway and inhibition of the apoptosis pathway. XIRP1 was mainly involved in the activation of the apoptosis and the EMT pathways and inhibition of the DNA damage and the PI3K pathways (Figures 7C,D).

Finally, the TIMER database was used to analyze the correlation between hub IRGs and immune cells. The results showed that these key genes were significantly correlated with the infiltration of CD4+ T cells, CD8+ T cells, macrophages, B cells, and neutrophils. Assuming that a correlation coefficient >0.3 was considered a strong correlation, further analysis showed that CLEC17A was positively correlated with the infiltration of B cells and CD4+ T cells. However, INHA was negatively correlated with the infiltration of CD8+ T and dendritic cells. However, there was no strong correlation between XIRP1 and the infiltration of immune cells (Figure 7E). Moreover, the relationship between copy number variation (CNV) of hub IRGs and immune cell infiltration was further analyzed. The results showed that there were significant differences between the CNV of these hub IRGs and immune cell infiltration. Arm-level deletion of the CLEC17A gene was closely related to the infiltration of B cells, CD4+ T cells, macrophages, neutrophils, and dendritic cells. Arm-level gain of the INHA gene was closely related to the infiltration of CD4+ T cells. Arm-level deletion of the XIRP1 gene was closely related to the infiltration of CD4+ T cells and macrophages (Figure 7F).

## Discussion

Tumor-infiltrating immune cells in the TME significantly contribute to the prognosis of lung adenocarcinoma. Therefore, it is essential to develop a TME-related immune prognostic model for appropriate clinical management of lung adenocarcinoma. Accordingly, the aim of this study was to develop a novel TME-related immune prognostic model for lung adenocarcinoma.

Although some studies have explored the prognostic value of TME-related IRGs in lung adenocarcinoma, certain key issues of these models remain to be resolved.

Yang et al. (18) used the CIBERSORT algorithm to analyze a TME-related prognostic immunity-based model for lung adenocarcinoma. This model was developed to evaluate the relative levels of the 22 immune cell phenotypes, primarily including B cells, T cells, macrophages, dendritic cells, plasma cells, natural killer cells, and mast cells. Moreover, this algorithm is primarily used to evaluate immune cells; however, it cannot be used to evaluate stromal cells in the TME. Yue et al. (19) used the ESTIMATE algorithm to investigate the TME-related immune prognostic characteristics of lung adenocarcinoma. However, they directly enumerated immune and stromal cells from their expression profiles of all genes expressed in lung adenocarcinoma and normal tissues. Moreover, differentially expressed IRGs were analyzed using Wilcoxon correlation analysis between tumor and normal tissue. However, some limitations are associated with the analysis of multi-dimensional tumor gene expression profiles through the Wilcoxon rank-sum test. These findings indicate that these TME-related prognostic immunity-based models have not been adequately evaluated. These issues can be resolved primarily by improving the algorithm of IRGs in the TME and to identify more specific TME-related IRGs for lung adenocarcinoma. Therefore, it is essential to develop a new TME-related immune prognostic model for lung adenocarcinoma.

To address these aforementioned limitations, in the present study, a new method was developed to identify differentially expressed IRGs. First, TME-related differentially expressed IRGs were identified exclusively from tumor samples by evaluating tumor-infiltrating immune cells and stromal cells via the ESTIMATE algorithm; this probably effectively reflected the TME-related IRGs in tumor tissue. Second, differentially expressed IRGs were analyzed on the basis of significant differences in OS between the high- and low-immune-score groups in terms of lung adenocarcinoma, rather than differences between lung adenocarcinoma and normal tissue using the Wilcoxon rank-sum test. The prognostic model, based on prognosis-associated differentially expressed genes, might more accurately predict the prognosis of lung adenocarcinoma. Finally, intersecting differentially expressed IRGs with significant prognostic characteristics in both immune and stromal scores were used for subsequent analysis. Both immune cells and stromal cells in each tumor sample were assessed, thus better reflecting the characteristics of the TME. Therefore, the TME-related immune prognostic model developed herein was different from those developed previously. In this study, we developed a more robust prognostic model of TME-related IRGs in lung adenocarcinoma.

Furthermore, this study shows that patients with lung adenocarcinoma and high immune scores had a better prognosis than those with low immune scores, which might be due to the involvement of upregulated IRGs in immune cell infiltration factors, such as cytokines and B cell immune pathways. Clinical studies have also shown that lung cancer patients with high immune infiltration of helper T cells have a better prognosis than those with low infiltration (20, 21). These findings were consistent with our results. Previous studies have suggested that IL-2 is involved in antitumor T cell infiltration, increasing the efficacy of immunotherapy (22). IL-33 also promotes myeloid-derived suppressor cells (MDSCs) and interferes with CD8+ T and natural killer (NK) cell infiltration (23). These studies have suggested that certain cytokines are involved in antitumor immune pathways, potentially elucidating the mechanisms associated with prognosis.

Moreover, GSEA was performed to further investigate the potential mechanism underlying the differences in prognosis between the two groups. The present results indicate that the immunoregulation and immune cell activation pathways are potentially associated with a better prognosis. The underlying putative mechanism potentially involves the enrichment of B and T cell immune pathways. Furthermore, the infiltration of these immune cells is associated with an enhanced prognosis among patients with lung adenocarcinoma. Our results are concurrent with those of the aforementioned studies.

Furthermore, this study described the functional prediction of potential hub IRGs. An enhanced understanding of these potential hub genes is essential to elucidate their mechanisms of action in the TME in lung adenocarcinoma. The present results suggest that although these genes were prognosis-related IRGs, they harbored different mutations involved in different pathways in lung adenocarcinoma, indicating their potential involvement in different immunoregulatory pathways in lung adenocarcinoma. Further analysis of the function of these genes revealed that these hub genes and their CNVs were different. Moreover, the association between these hub genes and the infiltration of B cells, CD4+ T cells, CD8+ T cells, neutrophils, and other immune cells was also different. These results indicate that different CNVs of these hub genes warrant further differentiation to better understand the association between hub genes and immune cell infiltration. Based on the aforementioned results, our results indicate that *CLEC17A, INHA*, and *XIRP1* are potential novel biomarkers for the prognosis of lung adenocarcinoma.

CLEC17A is a human lectin found in lymph node B cells and is involved in a variety of biological processes, including cell adhesion, intercellular interactions, and pathogen recognition (24). Previous studies have shown that CLEC17A is related to the B cell receptor signaling pathway and plays an important role in the pathogenesis of chronic lymphocytic leukemia (25). The present results further indicate that CLEC17A is associated with immune cell infiltration in lung adenocarcinoma, concurrent with previous reports. XIRP1 is a striated muscle protein and belongs to the Xin actin-binding repeat-containing protein (XIRP) family. Previous studies have shown that the XIRP1 gene is related to hypertension and nervous system development (26, 27). The function of the gene has not been reported in tumors. However, our study showed that it was not only related to the TME in lung adenocarcinoma but is also related to the prognosis of lung adenocarcinoma. INHA encodes a member of the transforming growth factor-beta (TGF-beta) superfamily of proteins. The function of the gene has not been reported in lung cancer. Our results showed that INHA was a marker of poor prognosis in lung adenocarcinoma. The possible mechanism was that INHA was involved in tumor angiogenesis, leading to tumor metastasis and poor prognosis (28). Further studies are required to elucidate the roles of these hub genes in the TME in the pathogenesis of lung adenocarcinoma.

This study also had some limitations. First, this study only mined data in the TCGA database and did not combine GEO database analysis. However, in our study, patient data were segregated into training and testing cohorts. In addition, the results were verified and analyzed in comprehensive TCGA and GSCALite datasets. Second, the function of the hub gene in our study was analyzed based on the TCGA database, and the validation function of the hub gene needs to be further confirmed by basic experiments. Constructing an immune-related prognosis model was the focus of our research; hence, there was no basic experiment on hub prognostic genes. Third, our study only analyzed the correlation of differentially expressed IRGs with immune cell infiltration, thus lacking the correlation analysis of the expression of PDL1 and tumor mutational burden.

## Conclusions

The robust TME-related immune prognostic model developed herein effectively predicted the prognosis of patients with lung adenocarcinoma, thus potentially guiding personalized treatment of lung adenocarcinoma in accordance with prognostic stratification. Further studies are required to elucidate the regulatory mechanisms of these IRGs in the TME and develop new treatment strategies.

## Data Availability Statement

Publicly available datasets were analyzed in this study. This data can be found here: https://portal.gdc.cancer.gov/.

## Author Contributions

XQ designed the study, analyzed the data, and drafted the paper. CQ critically revised it for important intellectual content. BQ and XK assisted in data acquisition and analysis. YH and WH revised the manuscript. All authors read and approved the final version of the manuscript.

## Conflict of Interest

The authors declare that the research was conducted in the absence of any commercial or financial relationships that could be construed as a potential conflict of interest.

## Supplementary Material

Supplementary Table 1 Immune-Stromal score of lung adenocarcinoma.

Supplementary Table 2 Differential IRGs estimated by immune score.

Supplementary Table 3 Differential IRGs estimated by stromal score.

Supplementary Table 4 Intersection of differential IRGs.

## Abbreviations

IRGs: immune-related genes
TME: tumor microenvironment
TCGA: The Cancer Genome Atlas data
DAVID: Database for Annotation, Visualization and Integrated Discovery.
